# And I hope you like jamming too

**DOI:** 10.1088/1367-2630/17/9/091001

**Published:** 2015-09-14

**Authors:** Dhananjay T Tambe, Jeffrey J Fredberg

**Affiliations:** 1 Department of Mechanical Engineering, Department of Pharmacology and Center for Lung Biology, University of South Alabama, Mobile, AL 36688, USA; 2 Harvard T.H. Chan School of Public Health, Boston, MA 02115, USA

**Keywords:** jamming, cancer, soft matter

## Abstract

The sorting of distinctly different cell types into specific tissue compartments has long been thought to be a problem in minimization of total free energy in immiscible fluids, wherein cell–cell adhesion, cell stiffness, and cell contraction combine to define an effective macroscopic tissue surface tension. Pawlizak *et al* (2015 *New J. Phys.* **17** 083049) now show not only that adhesion forces at interfaces unexpectedly fail to correlate with the density of adhesion molecules, but also that certain cancer cell lines unexpectedly fail to behave as a fluid, with cells becoming kinetically trapped in what might be a jammed, solid-like non-equilibrium state.

In his 1977 Reggae hit, the musician Bob Marley uses the song title ‘*Jamming*’ to mean getting together and then chilling out with like-minded friends or neighbors. Clearly, jamming is not something that you can do on your own. In the current issue of *NJP*, we learn from the new report of Pawlizak *et al* that cells too are predisposed to jam with like neighbors, at least in the problem of cell sorting as occurs in tissue development, tissue boundary formation, and cancer cell clustering [11]. In these fundamental biological processes, cell jamming has a remarkably similar connotation and, it now seems, previously unanticipated biological implications.

The problem of cell sorting has an illustrious history. By the 1740s, Trembley’s discovery of the regenerative potential of hydra had already rooted the idea that living beings are not ‘grown of their miniature version’ but are instead ‘gradually assembled from parts’ [7]. In the early 19th century, the focus progressively narrowed on the question of sorting of embryonic cells into anatomically correct structures. The goal was to identify the forces that drive this cell sorting. Some investigators considered properties that are attributable to each cell-type, such as adhesiveness, direction of movement, rate of motility, and time required to recover from a detachment event. By contrast, other investigators considered properties that are attributable to each region within the tissue aggregate, such as gradients of surface-tension, gradients of tissue contraction, localized mechanical forces, and localized water intake [3, 14].

In 1958, about two decades before the discovery of cadherins, Malcom Steinberg [12] proposed the differential adhesion hypothesis (DAH). Much as the tendency to minimize the total free energy associated with surface tension drives the dispersed droplets of immiscible fluids to merge into distinct but homogeneous fluid phases, so too the DAH in its original form proposes that the tendency to minimize total free energy associated with intercellular adhesion drives cell sorting into homogeneous tissue compartments [4]. Using this analogy, the DAH and its extensions attribute macroscopic tissue surface tension to the strength of intercellular adhesion, cell cortical tension and stiffness, and intercellular contraction [2, 5, 6, 9]. But even with these elaborations of the original DAH, the key underlying assumptions are that the tissue behaves dynamically as a fluid, and that the final sorted state corresponds to an equilibrium thermodynamic state, implying minimum free energy.

To assess validity of these assumptions, Pawlizak *et al* prepared spheroids from two of three cell lines: MCF-10A (from normal human mammary epithelium), MDA-MB-231 and MDA-MB-436 (from metastatic breast cancer). The cells in these spheroids indeed sorted into an end state that was reproducible. To assess if the end state is an equilibrium state, Pawlizak *et al* measured the cellular and cell–cell junctional properties implicated in the DAH, and other theories, and derived the corresponding sorted states. However, the experimentally observed sorted state and each of the theoretically derived sorted states did not match qualitatively.

Following this mismatch between the experimental and the theoretical sorted states, Pawlizak *et al* proposed that the cells within the aggregate might not be sampling all possible configurations randomly and ergodically, as do the constituents of a molecular fluid [9]. Instead of reaching an equilibrium thermodynamic state as predicted, the cellular collective could undergo kinetic arrest and thus find itself trapped in a jammed state. For example, MCF-10A cells formed compact aggregates with a smooth, rounded, fluid-like surface, whereas MDA-MB-436 and MDA-MB-231 cells formed aggregates with rough surfaces like sand grains, branches, protrusions and even holes (figure 1). Moreover, the reproducibility of the final sorted state suggests the unanticipated implications that cellular jamming might regulate structural organization of a tumor and control of cellular dissemination from a primary tumor.

**Figure 1.**
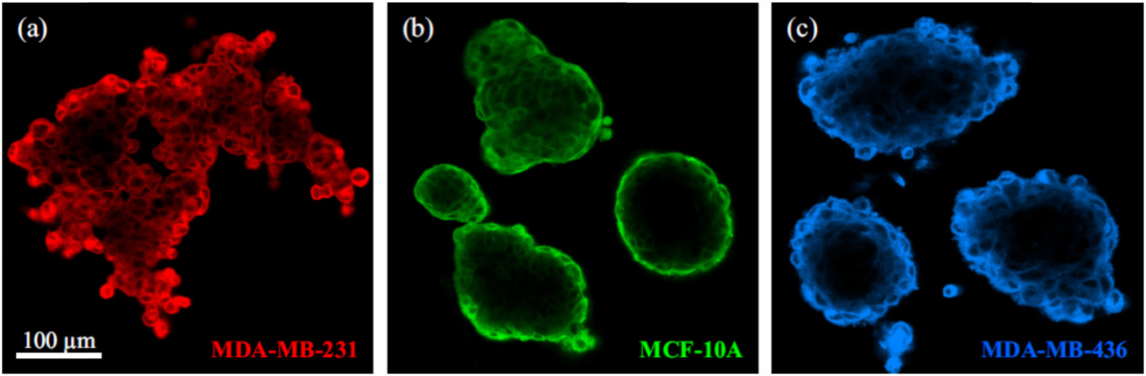
Multicellular clusters of certain cells types aggregate into smooth spheroids, as expected for a fluid-like state and minimization of surface free energy (b), whereas clusters of other cell types do not (a),(c), thus providing striking visual evidence that during cluster reorganization cells can become kinetically trapped—jammed—in a non-equilibrium solid-like state. From [11].

Many questions remain unanswered, however. In the crowded cellular environment of the tissue, the physical properties of each cell and its cell–cell junctions are strongly influenced by its immediate neighbors. As a consequence, for quantitative assessment of the final sorted state, the physical properties must be measured using multicellular assays such as monolayer stress microscopy or FRET probes [1, 13]. Use of these assays would overcome obvious questions about disparate physiological states of the cell when adhesion density is measured across the junction between a glass substrate and an isolated cell, intercellular adhesion is measured across the junction between an isolated cell pair, and cortical tension/stiffness is measured in a single isolated cell [11]. Moreover, use of multicellular assays will also provide a natural way to consider properties that emerge from the competition between intercellular adhesion and cortical tension and, in particular, changes of cell shape [8]. Cell shape bridges applicable length scales of jamming from molecules to tissue, and at the tissue scale has been recently found to provide a rigorous structural signature of jamming [10]. So, cells comprising the tissue do like to jam, and invitations are now open to investigate just how this comes to be.
